# Protective Effect of Huoxiang Zhengqi Oral Liquid on Intestinal Mucosal Mechanical Barrier of Rats with Postinfectious Irritable Bowel Syndrome Induced by Acetic Acid

**DOI:** 10.1155/2014/218383

**Published:** 2014-08-28

**Authors:** Yao Liu, Wei Liu, Qiu-Xian Peng, Jiang-Li Peng, Lin-Zhong Yu, Jian-Lan Hu

**Affiliations:** ^1^School of Traditional Chinese Medicine, Southern Medical University, Guangzhou 510515, China; ^2^New Drug Research and Development Center, Guangdong Institute of Traditional Chinese Medicine, Guangzhou 510520, China; ^3^Department of Biology, Hong Kong Baptist University, Kowloon Tong, Hong Kong; ^4^College of Pharmacy, Hunan University of Traditional Chinese Medicine, Changsha 410028, China

## Abstract

In this study, a rat model with acetic acid-induced PI-IBS was used to study the role of HXZQ oral liquid in repairing the colonic epithelial barrier and reducing intestinal permeability. Pathomorphism of colonic tissue, epithelial ultrastructure, DAO activity in serum, and the protein expression of ZO-1 and occludin were examined to investigate protective effect mechanisms of HXZQ on intestinal mucosa barrier and then present experimental support for its use for prevention and cure of PI-IBS.

## 1. Introduction

Postinfectious irritable bowel syndrome (PI-IBS) is a common disorder wherein symptoms of IBS begin after an episode of acute gastroenteritis. Published studies have reported incidence of PI-IBS that ranged between 5% and 32% [1]. Its high prevalence and the considerable effects on quality of life make PI-IBS a disease with high social cost. The majority of cases of PI-IBS meet the Rome II criteria for diarrhea-predominant irritable bowel syndrome (IBS-D), with predominantly loose stool passed more frequently than normal and with urgency [2]. The causes and underlying mechanisms of PI-IBS are still not fully understood, although it is believed that altered gut flora, changed intestinal permeability, activated gut immunity, and functional or structural changes in enteric nervous system are important factors [3–7]. Recent studies have suggested that increased intestinal permeability could be an important factor in the sequence of events leading to low-grade intestinal inflammation and disturbed bowel function [8, 9]. Intestinal epithelial permeability is regulated by a complex protein system comprising tight junction (TJ) and adherens junction proteins [10]. This increased permeability could be due to alterations of tight junction proteins. In addition, the changes of the ultrastructure of intestinal mucosal epithelial cells may be associated with the pathogenesis of PI-IBS [11]. To date, there are no successful treatments for PI-IBS, and management of PI-IBS primarily aims at relieving the symptoms [12, 13].

Natural medicine is generally considered safe and with few adverse drug reactions. As a representative natural medicine, traditional Chinese medicine (TCM) is more and more widely used nowadays. HXZQ oral liquid is a Chinese patent medicine composed of 10 kinds of Chinese medical medicines. It was modified based on a traditional formula named Huoxiang Zhengqi powder, which originated from the ancient Chinese pharmacy book “Formularies of the Bureau of the People's Welfare Pharmacies” (Taiping Huimin Hejiju Fang) in the Song Dynasty of China; it has been mainly used to treat gastrointestinal diseases for more than 1000 years. Clinical researches have proved that HXZQ has definite clinical effect for treating IBS-D [14]. Pharmacological research has shown that HXZQ can regulate gastrointestinal motility in a two-way manner, alleviates visceral hypersensibility, and has the effect of anti-inflammation and immune regulation [15–18].

At present, researches about the mechanism of HXZQ used in the treatment of IBS focus on gastrointestinal hormone, gastrointestinal dynamics, visceral sensitivity, nerve-endocrine-immune network and water metabolism [19, 20]. However, there is no systematic study about the efficacy of HXZQ in PI-IBS and whether it could regulate intestinal mucosal barrier function on PI-IBS is unknown. Therefore, in this study, a rat model with acetic acid-induced PI-IBS was used to study the role of HXZQ oral liquid in repairing the colonic epithelial barrier and reducing intestinal permeability. Pathomorphism of colonic tissue, epithelial ultrastructure, DAO activity in serum, and the protein expression of ZO-1 and occludin was examined to investigate protective effect mechanisms of HXZQ on intestinal mucosa barrier and then present experimental support for its use for prevention and cure of PI-IBS.

## 2. Materials and Methods

### 2.1. HXZQ Oral Liquid

Huoxiang Zhengqi oral liquid was purchased from Taiji Group Chongqing Fuling Pharmaceutical Co., Ltd. (Approve number: Z50020409, Lot number: 13051224).

HXZQ oral liquid consists of 13 herbs, including 80 g each of Atractylodis Rhizoma, Citri Reticulatae Pericarpium, Magnoliae Officinalis Cortex, and Pinelliae Rhizoma; 120 g each of Angelicae Dahuricae Radix, Poria, and Arecae Pericarpium; 10 g Licorice extract, 0.8 mL Patchouli oil, 0.4 mL volatile oil in Perillae Folium (Table 1). HXZQ oral liquid is manufactured by Taiji Group Chongqing Fuling Pharmaceutical Co. (Chongqing, China) following the approved Good Manufacturing Practice of the China's State Food and Drug Administration, according to criteria of Chinese pharmacopeia 2010.

Preparation: Magnoliae Officinalis Cortex was extracted by refluxing with 60% ethanol for 1 h, and, after filtering, ethanol extract was reserved; Atractylodis Rhizoma, Citri Reticulatae Pericarpium, and Angelicae Dahuricae Radix were distilled with water; the distillation was filtered and reserved. Arecae Pericarpium was decocted twice with water, and then the extraction solutions were combined to be filtered. After Poria was boiling, it was warm soaked two times at 80°C and then filtered. After raw Arecae Pericarpium was immersed thoroughly in water, plus Zingiberis Rhizoma (6.8 g), it was decocted twice with water and filtered. The above filtrates were merged and then concentrated to obtain the clear cream (relative density: 1.10–1.20, 50°C). Licorice extract was added to the clear cream. After being mixed well, the clear cream was precipitated with ethanol (2 times of the amount of clear cream) and filtered. Filtrate merged with ethanol extract of Magnoliae Officinalis Cortex; then ethanol was recycled. Patchouli oil, Patchouli oil, Volatile oil in Perillae Folium and the above distillate were mixed well, and add water to make the whole amount to 1025 ml. After the pH value was adjusted to 5.8–6.2 with sodium hydroxide solution, it was let stand, filtered, encapsulated, and sterilized.

### 2.2. Quantitative Analysis of HXZQ Oral Liquid Using Ultra High Performance Liquid Chromatography

The reference standard stock solutions of liquiritin, aurantiamarin, ammonium glycyrrhetate, honokiol, and magnolol (0.5 mg/mL) were prepared in methanol and stored at <4°C. The standard solutions were prepared using five concentrations of diluted solutions (methanol). All the calibration curves were generated by assessing the peak areas for the five concentrations in the range of 0.0017~0.128 *μ*g for all standard samples. The linearity of the peak area (*y*) versus concentration (*x*, *μ*g/mL) curve for each component was used to calculate the contents of the main components in HXZQ oral liquid. Quantitative analysis was performed under concurrent conditions using an 1290 infinity high-performance liquid chromatography (UPLC; Agilent Technologies) equipped with an autosampler (G1367E), column oven (G1316C), binary pump (G4220A), and DAD (G4212A). The analytical column comprised a Zorbax RRHD Eclipse C18 (2.1 × 100 mm; particle size, 1.8 *μ*m) and was maintained at 30°C. The data were acquired and processed by chemstation software (Agilent Technologies). The mobile phase comprised acetonitrile (A) with 0.05% phosphate acid (B). The gradient flow was as follows: 0–5 min, 17–20% A; 5–12 min, 20–50% A; 12–18 min, 50–70% A; 18-18.5 min, 70–95% A; 18.5-23.5 min, 95% A. The analysis was carried out at a flow rate of 0.6 mL/min, and detection was performed at a wavelength of 220 nm. The injection volume was 1 *μ*L.

### 2.3. Positive Control Drug

Trimebutine maleate tablets were purchased from Kaikai Yuansheng Medicine Co., Ltd. (Lot number: 13032817).

### 2.4. Animals

Sprague-Dawley (SD) rats (250–270 g, 2 months old) were purchased from the Laboratory Animal Centre of Southern Medical University (License: SCXK Guangdong 2006-0015) and were acclimated in the facility for 1 week before being used in the experiments. The animals were housed at 25 ± 1°C with a 12:12 h light/dark cycle (lights on at 7:00 a.m.) and were allowed to access water and food. All procedures in this study were conducted in accordance with the NIH Guide for the Care and Use of Laboratory Animals, and the experiments were approved by the Laboratory Animal Ethics Committee of Southern Medical University.

### 2.5. Experimental Design

#### 2.5.1. Grouping and Administration

The rats were randomly divided into six groups including normal control group, PI-IBS model group, trimebutine maleate tablet group (TMT group), Huoxiang Zhengqi low-dose group (low-dose of HXZQ), middle-dose group (middle-dose of HXZQ), and high dose group (high-dose of HXZQ) with 10 rats in each group and treated with water (control group and PI-IBS model group), trimebutine maleate tablets (0.1 g/kg/d, TMT group), and HXZQ oral liquid (1.7, 3.3, and 6.6 mL/kg/d, low-dose, middle-dose, and high dose of HXZQ). Administration dose of HXZQ oral liquid was established according to the usage and dosage criteria of Chinese pharmacopeia 2010. At the seventh day after acetic acid administration, agent was administered to each group through intragastric administration for 5 days.

#### 2.5.2. PI-IBS Animal Model Establishment

The PI-IBS rat model was developed according to previous report [21]. After an overnight fast, the rats were anesthetized with ether, and a plastic catheter (external diameter = 0.96 mm) was inserted into the descending colon at the depth of 8 cm from anus; then acetic acid (4%, 1 mL/rat) aqueous solution was instilled slowly for 30 s. Then, 1 mL phosphate buffered saline (PBS) was instilled to dilute the acetic acid and flush the colon. The normal control group animals were handled identically except that 1 mL saline was instilled instead of 4% acetic acid.

#### 2.5.3. Sample Collection

On the day after the last treatment, the rats were sacrificed. A 6 cm long proximal colon (1-2 cm from caecum) was harvested and divided into 3 parts. The proximal was fixed in 4% paraformaldehyde and embedded in paraffin for histopathological observation; then immunohistochemical detection was conducted to observe the expression of ZO-1 and occludin; the middle part was collected for microscopic evaluation by TEM; the distal part was proceeded for Western blotting analysis. Abdominal aortic blood was collected for DAO assay in serum.

#### 2.5.4. Histological Examination of Colon Tissue

A 1 cm section of fresh colon tissues were fixed in 4% paraform for 12 h, then dehydrated in 50%, 60%, 70%, 80%, 90%, and 100% alcohol separately, and embedded in paraffin at 56–60°C. The samples were cut into sections (4 *μ*m for each), deparaffinized in dimethyl benzene, gradiently dehydrated in alcohol and stained with hematoxylin, eosin (H&E), dehydrated in 70%, 90%, and 95% ethanol, and finally cleared in xylene. Pathological histology slice of colonic tissue was observed under a light microscope (original magnification ×400).

#### 2.5.5. Ultrastructural Observation by TEM

The colon segments were cut into 1 mm^3^ strips, fixed for 4 h at 4°C in 3% glutaraldehyde, fully washed for 3 times in 0.1 mol/L PBS, postfixed for 2 h at 4°C in 2% osmium tetroxide, dehydrated in a graded series of ethanols, embedded in Epon 812, cut into ultrathin sections (75 nm), and then stained with uranyl acetate and lead citrate. Sections were viewed in a HITACHI H-600 electron microscope at 60 kV (HITACHI, Tokyo, Japan).

#### 2.5.6. ELISA Assay of DAO

Serum of abdominal aortic blood was separated and stored at −80°C until use. DAO level was determined using ELISA kits according to the protocol as described previously [22].

#### 2.5.7. Immunohistochemistry for ZO-1 and Occludin Expression

The tissue sections were rinsed in PBS (pH.7.4) and placed in polycarbonate staining jars (Kartell, Italy) filled with 10 mM sodium citrate buffer solution (pH.6.0). After being exposed to microwave for 15 min to retrieve antigen, then the tissues were incubated in a solution of 3% bovine serum albumin, 0.5% Triton X-100 in PBS. Sections were incubated with rabbit anti-zo-1 antibody (1 : 200, Millipore) and rabbit anti-occludin antibody (1 : 200, abcam), respectively, in PBS containing 0.5% Triton X-100 overnight at 4°C. After rinsed in PBS, secondary antibody (polyperoxidase rabbit IgG, Zhongshan Biotechnology Co. Ltd.) was incubated for 2 h at room temperature, and then rinsed and cover-slipped. Sections were observed with a microscope (Nikon 80, Japan), captured with 400x, and analyzed using Image-Pro Plus 6.0 software. The optical densities in colonic mucosa from 8 random fields per section were calculated.

#### 2.5.8. Western Blotting for ZO-1 and Occludin Expression

The colonic tissues were homogenized and sonicated on ice for protein extraction. After the protein was quantified, lysates were denatured at 100°C for 5 min. An equal amount of protein (30 mg/well) was electrophoresed on 10% SDS-PAGE gel. The proteins were then electroblotted to PVDF membranes (Bio-Rad) and blocked with 5% nonfat skim milk for 1 h. Subsequently, the blot was incubated in rabbit anti-ZO-1 and rabbit anti-occludin antibody (1 : 1000, Santa Cruz, CA) supplemented with 2% nonfat skim milk for 2 h at room temperature. After washing, the membrane was incubated in horseradish peroxidase- (HRP-) conjugated secondary goat anti- rabbit antibodies (1 : 5000, Zymed Laboratories) in 2% nonfat skim milk for an hour at room temperature. Bands on the membranes were visualized using ECL (Amersham, UK). Distinct immunoreaction products were revealed and images of the bands were developed on film (Biomax X-ray film, Kodak). To check the amount of protein loaded, the immunoblots were treated with stripping solution (62.5 mM Tris buffer, pH 6.7, containing 2% SDS and 100 mM b-mercaptoethanol) for 30 min at 50°C and incubated with mouse monoclonal anti-*β*-actin antibody (Sigma) followed by horseradish peroxidase-coupled goat anti-mouse IgG (Pierce). Optical densities of the individual bands were scanned and quantified using NIH Image J. All detected protein bands were normalized to *β*-action levels.

### 2.6. Statistical Analysis

Statistical analysis was performed using SPSS 13.0 software. Data were expressed as the mean ± standard error (SE). One-way ANOVA tests were performed to assess the differences in the cytokine expression levels. A post-hoc Bonferroni test was used to assess the differences between the individual groups. *P* < 0.05 was considered significant.

## 3. Results

### 3.1. Fingerprint Analysis of HXZQ Oral Liquid

The standard curves for the five components containing liquiritin, aurantiamarin, ammonium glycyrrhetate, honokiol, and magnolol were *y* = 36251*x* − 15.336 (*r* = 0.9999), *y* = 26551*x* − 11.451 (*r* = 1.000), *y* = 2488*x* − 11.552 (*r* = 0.9999), *y* = 81440*x* − 51.221 (*r* = 0.9999), and *y* = 91221*x* − 11.635 (*r* = 0.9993), respectively. The components obtained from UPLC analysis of HXZQ oral liquid and standard mixtures were detected at 220 nm. The retention times of each component were 2.52 min (liquiritin), 5.02 min (aurantiamarin), 10.85 min (ammonium glycyrrhetate), 14.02 min (honokiol), and 15.32 min (magnolol). The contents of each component were in the range of 0.045~0.66 g/L (Table 2).

### 3.2. Histopathology of Colonic Tissues

According to the observation, the structure of the intestinal mucosa in control group was intact, the epithelium was normal, simple columnar epithelium and enteraden in colonic mucosa were ordered arrangement, and goblet cells were rich. A small amount of plasma cells within the interstitial were observed. In low-dose of HXZQ, middle-dose of HXZQ, and high-dose of HXZQ groups, histological features were in accord with control group, and no overt inflammatory infiltration of neutrophils in the lamina propria and interstitial edema was observed. However, epithelial cell sloughing off and few neutrophil infiltrations were observed in PI-IBS model group.

### 3.3. Ultrastructure of Rat Colonic Epithelial Tissue

**Figure 1:**
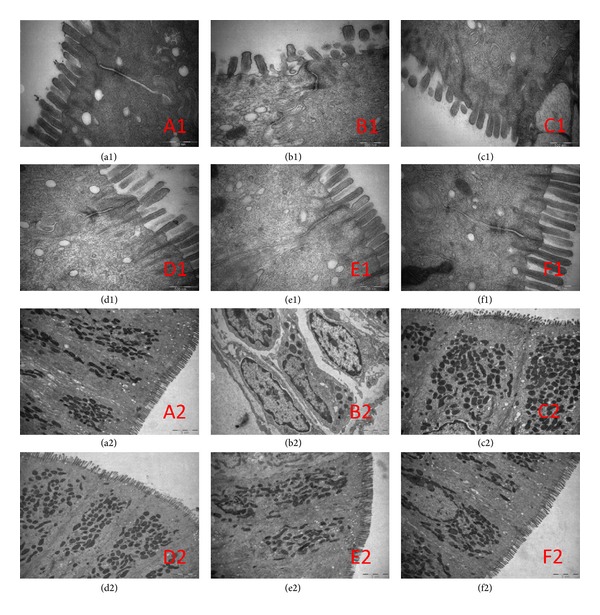
Ultrastructure changes of colonic epithelial samples from rats with PI-IBS under transmission electron microscope (12000x). Original magnification 400x. Normal control rats ((a1), (a2)), PI-IBS model rats ((b1), (b2)), and TMT treated rats ((c1), (c2)), low-dose of HXZQ rats ((d1), (d2)), middle-dose of HXZQ treated rats ((e1), (e2)), and high-dose of HXZQ rats ((f1), (f2)) ((a1)–(f1), 70000x; (a2)–(f2), 12000x).

In control group, rat intestinal epithelial cell microvilli were arranged in neat rows. The epithelial cell membrane and the nuclear membrane were integral. The nucleolus could be seen clearly. In the cytoplasm, both the endoplasmic reticulum and mitochondria could be seen clearly. The junction of the epithelial cells was tight (Figure 1 (a1), (a2)). However, in PI-IBS model group, the microvilli on the surface of epithelial cells of the intestine were sparse or distributed irregularly with different lengths. There was degeneration or necrosis in a few intestinal epithelial cells. The junctions among the cells were not broadened obviously. In addition, mast cells were filled with high-density particles and some vacuoles in cytoplasm and showed active degranulation state (Figure 1 (b1), (b2)). In TMT group, microvilli showed slight dropout and tight junctions were unclear in a few of the epithelial cells (Figure 1 (c1), (c2)). In low-dose of HXZQ, middle-dose of HXZQ, and high-dose of HXZQ groups, the epithelial cell membranes were intact, the cellular shapes were overall normal, and the regular distribution of microvilli were observed. To conclude, the colonic epithelial ultrastructure was better maintained compared to PI-IBS model group (Figure 1).

### 3.4. Diamine Oxidase (DAO) Activity

**Figure 2:**
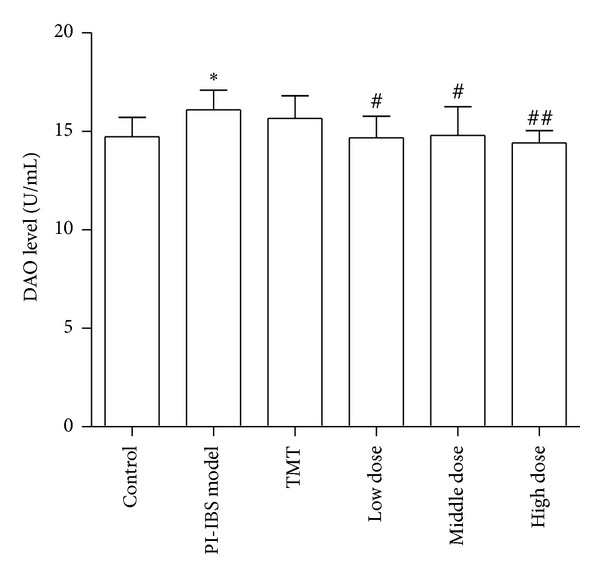
ELISA analysis of DAO in serum from PI-IBS rat. *P < 0.05 versus normal control group; ^#^ *P* < 0.05, ^##^ *P* < 0.01 versus PI-IBS model group.

Figure 2 shows that the levels of serum DAO activity in PI-IBS model group increased significantly compared with that in low-dose of HXZQ, middle-dose of HXZQ, and high-dose of HXZQ groups (14.67 ± 1.09, 14.79 ± 1.45, and 14.41 ± 0.63 versus 16.03 ± 1.01, **P < 0.01, *P < 0.05). However, compared with control group, no significant difference in serum levels of DAO among other groups was observed.

### 3.5. The Tight Junction Protein ZO-1 and Occludin Expression in the Colonic Mucosa

**Figure 3:**
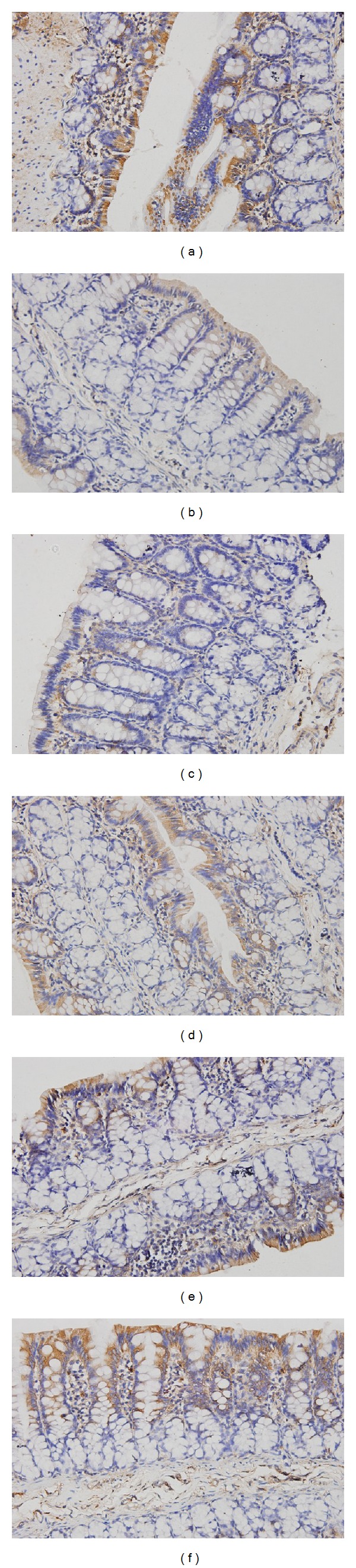
Immunohistochemical analysis of occludin in colonic mucosa from rats with PI-IBS. Original magnification 400x. Normal control rats (a), PI-IBS model rats (b), and TMT treated rats (c), low-dose of HXZQ rats (d), middle-dose of HXZQ treated rats (e), and high-dose of HXZQ rats (f). Semiquantitative analysis of occludin-staine areas showed a significant decrease in rats with PI-IBS, and HXZQ could significantly increase these areas.

**Figure 4:**
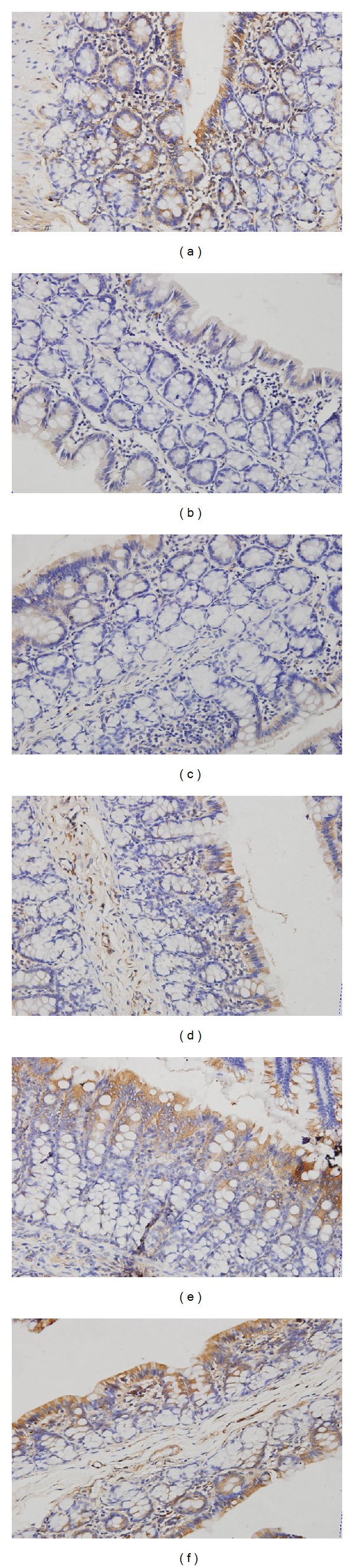
Immunohistochemical analysis of ZO-1 in colonic mucosa from rats with PI-IBS. Original magnification 400x. Normal control rats (a), PI-IBS model rats (b), and TMT treated rats (c), low-dose of HXZQ rats (d) rats, middle-dose of HXZQ treated rats (e), and high-dose of HXZQ rats (f). Semiquantitative analysis of ZO-1-staine areas showed a significant decrease in rats with PI-IBS, and HXZQ could significantly increase these areas.

Immunohistochemistry results showed that in the control group, the proteins expression of ZO-1 and occludin were evenly distributed on the edges of intestinal epithelial cells with honeycomb or linear shapes. In PI-IBS model group, ZO-1 and occludin staining was unevenly distributed or faded, and the proteins expression of ZO-1 and occludin were dramatically reduced. However, after being treated with HXZQ oral liquid, the downregulated ZO-1 and occludin protein expressions heightened significantly (Figures 3 and 4). As shown in Table 3, the average IHC optical density values of ZO-1 and occludin decreased significantly (*P* < 0.01) in PI-IBS model group, as compared with control group. Nevertheless, the values of ZO-1 and occludin in low-dose of HXZQ group, middle-dose of HXZQ group, and high-dose of HXZQ group were significantly higher than PI-IBS model group (*P* < 0.01). Difference of ZO-1 and occludin IHC average optical density values between PI-IBS model group and TMT group was not statistically significant.

**Figure 5:**
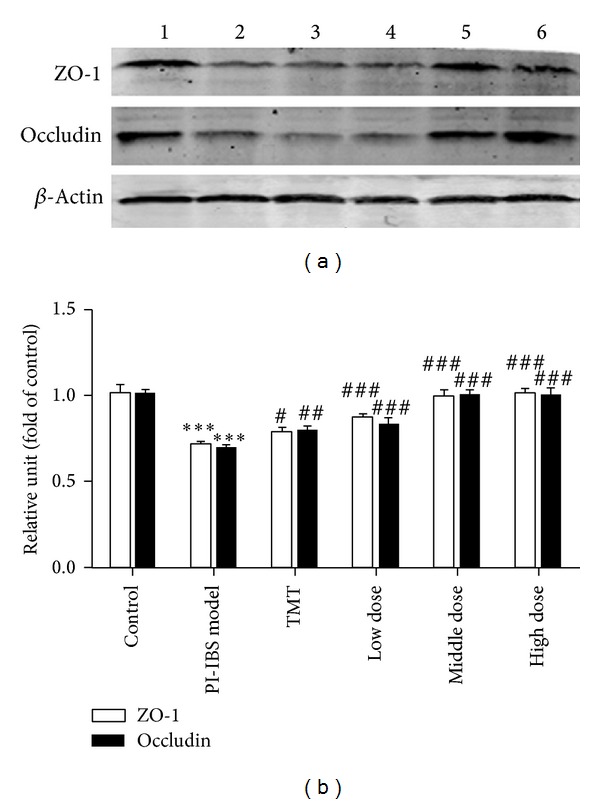
Expression of ZO-1 and occludin proteins in PI-IBS rats. Normal control rats (lane 1), PI-IBS model rats (lane 2), TMT treated rats (lane 3), low-dose of HXZQ treated rats (lane 4), middle-dose of HXZQ treated rats (lane 5), and high-dose of HXZQ treated rats. Semiquantitative analysis of ZO-1 and occludin showed a significant decrease in the level of ZO-1 and occluding in PI-IBS rat and markedly increased in HXZQ treated rats. *P < 0.05, **P < 0.01 versus the control group; ^###^ *P* < 0.001, versus PI-IBS model group.

Given the important role of the ZO-1 and occludin in tight junction, ZO-1 and occludin expression was further evaluated by Western blotting. As shown in Figure 5, compared with the control group (100%), the ZO-1 and occludin protein expression decreased significantly (*P* < 0.001) in PI-IBS model group. Besides, the protein expression of ZO-1 and occludin in middle-dose of HXZQ and high-dose of HXZQ group was significantly higher than those in group PI-IBS model group and TMT group (*P* < 0.001).

## 4. Discussion

In recent years, the role of intestinal infection in PI-IBS pathogenesis has attracted increasing attention. Intestinal infection can damage intestinal mucosal barrier and make intestinal mucosal epithelial permeability increased and the number of mast cells and cytokines increased, which affect the visceral sensation, gastrointestinal motility, and gastrointestinal hormone secretion [23–25]. Intestinal mucosa barrier damage with different degrees has existed in PI-IBS patients. HXZQ oral liquid can regulate the function of gastrointestinal tract and has definite curative effect for treating IBS-D. The aim of this study is to interpret protective effect of HXZQ oral liquid on intestinal mucosal mechanical barrier of rats with PI-IBS.

Currently, postinflammatory IBS models induced by chemical agents, such as acetic acid, dextran sulfate sodium, deoxycholic acid, mustard oil, zymosan, and trinitrobenzene sulfonic acid (TNBS), have been widely used in mechanistic studies of PI-IBS. TNBS-induced postinflammatory model is commonly used as PI-IBS model. However, certain aspects of the protocols of the model are not standardized, such as the dosage of TNBS, the depth of TNBS administration, and the time point for evaluating the model [26]. In this study, the rat model of intracolonic instillation of acetic acid-induced colitis (4%) showed major features of PI-IBS after subsidence of the acute inflammation in colon. The advantages of this model include not only a relatively short period for the subsidence of inflammation but also ease of access to the colon. In this study, PI-IBS model group animals have been shown loose stools and marked visceral hypersensitivity with no evidence of histological inflammation. All the observations suggested that the features of PI-IBS could be identified in this model. Meanwhile, the effect of HXZQ oral liquid on basic symptoms associated with PI-IBS was studied by measuring water ratio of rats' faeces, defecation time, and intestinal sensitivity. According to the results, HXZQ oral liquid could significantly reduce water ratio of rats' faeces, prolong defecation time, relieve loose stools symptom, and alleviate visceral hypersensitivity (Supplementary data). It is showed that the effects of above factors of HXZQ were similar to TMT.

A recent review revealed that the mechanisms of kampo medicines including some components of HXZQ were involved in improving gastric dysmotility mediated by NO or 5-HT3 receptor pathways and anti-inflammatory, and so on [27]. Traditionally to test the permeability of the small intestinal mucosa, the DAO activity is detected due to the fact that DAO in layer of villi of the intestinal mucosa reflects the function and structure of the small intestine and its plasma level can be used as a marker for evaluation of maturation and integrity of the intestinal mucosa. DAO activities or levels in serum are very low in normal conditions [28, 29], but when the intestinal mucosal epithelial cells are damaged, DAO will be released, and large amount of DAO will enter the systemic circulation [30]. DAO is an intracellular enzyme with high activity existing in intestinal villous cells in mammalians, especially high in the jejunum and ileum [31]. The activity of DAO in intestinal mucosa decreases when the epithelium are injured; thus DAO activity of intestinal mucosa can indicate the changes in its cellular integrity. In this study, the serum DAO levels of PI-IBS model group were significantly increased compared with those in HXZQ groups. No significant difference of serum DAO levels between HXZQ groups and NC group was detected. Our results demonstrated that small intestinal mucosal permeability of the rats with PI-IBS was increased slightly. Therefore, the protective effect of HXZQ on colonic mucosa in PI-IBS rats may be caused by reducing the intestinal mucosal permeability and/or improving the integrity of the intestinal mucosa. Moreover, the histomorphological change of colon mucosa was not observed in PI-IBS rats, but the changes of the ultrastructure of cells have been observed. For example, the microvilli distributed unevenly with various lengths, and there was degeneration or necrosis in few intestinal epithelial cells. These observations indicated that intestinal mucosa mechanical barrier of PI-IBS rats was impaired and intestinal permeability was increased. After administration of HXZQ, the ultrastructure of mucosal epithelial cell was improved, which demonstrates that HXZQ can repair the ultrastructure of the damaged cell, decrease the permeability, and inhibit inflammatory reaction, thus improving the intestinal mucosal barrier function.

The intestinal barrier function was maintained by a series of epithelial TJs, protecting the body from pathogens and other toxic luminal substances. Transmembrane proteins (such as occludin, tricellulin, claudins, and junctional adhesion molecule) interact with cytoplasmic peripheral membrane proteins (such as ZO-1, ZO-2, ZO-3, and cingulin) that constitute the frames of tight junctions [32]. Recent studies have proved that increased permeability of the tight junctions is thought to contribute to PI-IBS [25, 33]. The tight junction seals the space between adjacent epithelial cells, and, in intact gastrointestinal epithelia, tight junction permeability is the rate-limiting step that defines the overall epithelial permeability [34]. ZO-1 and occludin are the key proteins forming the tight junctions, and their role in barrier permeability has been thoroughly examined and found crucial for the regulation of tight junction [35, 36]. Our results have shown that the expression of ZO-1 and occludin protein in the colonic mucosa of PI-IBS rats was significantly increased after administrating HXZQ. Therefore, we speculate that the ultrastructural change is linked to the degradation of ZO-1 and occludin and the subsequent increase in paracellular permeability. Meanwhile, our results suggest that alteration of tight junction proteins may be involved in the initiation of PI-IBS and HXZQ can upregulate the expression of ZO-1 and occludin protein, which suggests that the tight junction afforded by occludin and ZO-1 is important in maintaining the epithelial barrier integrity in response to HXZQ.

In the study, trimebutine maleate was used as the positive control drug. Trimebutine maleate, a weak *μ*-opioid receptor agonist, has been widely used for the treatment of irritable bowel syndrome to relieve abdominal pain and alter bowel habits [37, 38]. Moreover, trimebutine maleate is effective in reducing the colonic muscle hypercontractility of PI-IBS mice [39]. Our results showed that trimebutine maleate had no obvious effect on improving structure and function of intestinal mucosal mechanical barrier in PI-IBS rats. Thus, as is shown in the previous research reports, trimebutine maleate pharmacological mechanisms mainly reflected in regulating the hypo- and hypermotility of the broad gastrointestinal tract with a dual action depending on the enkephaline receptor subtype [40].

## 5. Conclusions

The purpose of this study is to explore the effect and the protective mechanism of HXZQ oral liquid on intestinal mucosal mechanical barrier in acetic acid-induced PI-IBS rats. The results showed that the HXZQ can improve the ultrastructure of intestinal epithelial cells, upregulate the expression of ZO-1 and occludin, and downregulate activity of DAO in peripheral blood serum. Overall, our findings provide the novel mechanisms by which HXZQ oral liquid can repair and enhance the function of intestinal mucosal mechanical barrier, which implies that HXZQ can be a potential drug for PI-IBS patients.

## Supplementary Material

S1. Measurement of water ratio of rats' faeces.
S2. Assessment of intestinal sensitivity.

## Figures and Tables

**Table 1:** The herbal prescription of HXZQ oral liquid.

Herbal name	Scientific name	Local name	Place of origin	Relative amounts
Atractylodis Rhizoma	*Atractylodes lancea* (Thunb.) DC. or *Atractylodes chinensis* (DC.) Koidz.	Cangzhu	China (Jiangsu)	80 g
Citri Reticulatae Pericarpium	*Citrus reticulata* Blanco	Chenpi	China (Sichuan)	80 g
Magnoliae Officinalis Cortex	*Magnolia officinalis* Rehd. et Wils. or M*agnolia officinalis* Rehd. et Wils. var. *biloba* Rehd. et Wils.	Houpo	China (Sichuan)	80 g
Angelicae Dahuricae Radix	*Angelica dahurica* (Fisch. ex Hoffm.) Benth. et Hook. f. or A*ngelica dahurica* (Fisch. ex Hoffm.) Benth. et Hook. f. var. *formosana* (Boiss.) Shan et Yuan	Baizhi	China (Sichuan)	120 g
Poria	*Poria cocos* (Schw.) Wolf	Fuling	China (Yunnan)	120 g
Arecae Pericarpium	*Areca catechu* L.	Dafupi	China (Hainan)	120 g
Pinelliae Rhizoma	*Pinellia ternata* (Thunb.) Breit.	Banxia	China (Henan)	80 g
Licorice extract	*Glycyrrhiza uralensis* Fisch. or *Glycyrrhiza inflate* Bat. or *Glycyrrhiza glabra* L.	Gancao Jingao	China (Neimenggu)	10 g
Patchouli oil	*Pogostemon cablin* (Blanco) Benth.	Guanghuoxiang You	China (Guangdong)	0.8 mL
Volatile oil in Perillae Folium	*Perilla frutescens* (L.) Britt.	Zisuye You	China (Sichuan)	0.4 mL

**Table 2:** Quantitative analysis of HXZQ oral liquid and its reference compounds.

Compound	Retention time (min)	Mean (g/L)	SD
Liquiritin	2.52	0.045	3.53
Aurantiamarin	5.02	0.18	5.52
Ammonium glycyrrhetate	10.85	0.66	1.05
Honokiol	14.02	0.19	3.33
Magnolol	15.32	0.20	2.09

**Table 3:** Comparison of ZO-1 and occludin IHC average optical density values among the six groups (*n* = 8).

Group	ZO-1	Occludin
Control	158821.4 ± 75669.8	167713.9 ± 69214.7
PI-IBS model	90791.4 ± 25604.2*	118096.9 ± 30682.4∗∗
TMT	134687.3 ± 52725.6	147252.4 ± 72804.5
Low-dose of HXZQ	165330.2 ± 58489.6^###^	214315.1 ± 54218.04^###^
Middle-dose of HXZQ	195346.5 ± 60506.4^###^	232473.4 ± 73962.4^###^
High-dose of HXZQ	206713.8 ± 41338.6^###^	221420.3 ± 81806.4^###^

Values given as mean ± standard deviation. *P < 0.05, **P < 0.01 versus control group; ^###^ *P* < 0.001 versus PI-IBS model group.
