# Supplementary material for: Protective Effect of Huoxiang Zhengqi Oral Liquid on Intestinal Mucosal Mechanical Barrier of Rats with Postinfectious Irritable Bowel Syndrome Induced by Acetic Acid
Source: Evid Based Complement Alternat Med. 2014 Aug 28;2014:218383. doi: 10.1155/2014/218383 (PMC4164314; doi:10.1155/2014/218383)
Supplement: Supplementary file 1 — S1. Measurement of water ratio of rats' faeces. S2. Assessment of intestinal sensitivity. [file 218383.f1.docx]

**Supplementary data**

S1. Measurement of [water](app:\ds\water) [ratio](app:\ds\content) of rats’ [faeces](app:\ds\faeces)

Method:

[Water](app:\ds\water) [ratio](app:\ds\content) of rats’ [faeces](app:\ds\faeces) in each groups was measured at the end of animal model preparation (the seventh day) and after the treatment (the twelfth day). Fasting for 12 h, animal forelimbs were bound by cotton tape for 3h, and rats feces was collected in the period of restraint stress. Wet weight and dry weight of feces were weighed respectively, and then [water](app:\ds\water) [ratio](app:\ds\content) of rats’ [faeces](app:\ds\faeces) was calculated by (Wet weight - dry weight) / wet weight ×100%

Result:

As shown in Table 1, on the seventh day, [water](app:\ds\water) [content](app:\ds\content) in rats’ [faeces](app:\ds\faeces) of PI-IBS model group, TMT group and HXZQ treatment groups were higher than that of normal control group (P<0.001). On the twelfth day, [water](app:\ds\water) [content](app:\ds\content) in rats’ [faeces](app:\ds\faeces) of PI-IBS model group was higher than that of normal control group (P< 0.001), [water](app:\ds\water) [content](app:\ds\content) in rats’ [faeces](app:\ds\faeces) of TMT group and HXZQ treatment groups were lower than PI-IBS model group (P<0.05, P<0.001), meanwhile there was no statistically significant difference between middle-dose and high-dose groups of HXZQ with normal control group.

Table 1 The [water](app:\ds\water) [ratio](app:\ds\content) of rats’ [faeces](app:\ds\faeces) in each groups（n=8）

| Group | The seventh day（％） | The twelfth day（％） |
| --- | --- | --- |
| control | 48.84±2.95 | 47.18±5.51 |
| PI-IBS model | 65.00±4.00*** | 59.49±5.67*** |
| TMT | 63.19±3.76*** | 53.69±4.08**# |
| low-dose of HXZQ | 64.38±2.86*** | 54.78±3.05**# |
| middle-dose of HXZQ | 64.84±3.12*** | 49.49±2.89### |
| high-dose of HXZQ | 63.65±2.24*** | 49.74±3.78### |

Values given as mean±standard deviation.***P*<0.01，****P*<0.001 vs. control group； #*P*<0.05，###*P*<0.001 vs. PI-IBS model group.

S2. Assessment of intestinal sensitivity

Method:

The intestinal sensitivity to colorectal distention(CRD) were assessed in all groups on the 7th day 12th day after intracolonic instillation of acetic acid by observing abdominal withdrawal reflex. Fasting for 12 h, a catheter (Braun 8F) coated with paraffin oil was inserted into the descending colon at the depth of 2 cm from anus. Attaching catheter to the rat’s tail by medical tape was to prevent the catheter sliding out. The rats were then housed in small Lucite cubicles (20 cm×8 cm×8 cm) which limited rats’ various activities, but abdominal contraction reflection can be observed. When rats were in quiet state, physiological saline (26℃~28℃) was injected into balloon catheter slowly to resulted in colonic distention. The [minimum](app:\\ds\\minimum) of injected water volume, which induced abdomen lifted (abdomen is apart from the horizontal plane about 0.2 cm) was recorded. The liquid in balloon catheter quickly drawed out after the completion of CRD on every time. Measurement was repeated three times on the same rat with interval of 15 min. The [mean](app:\\ds\\mean) [value](app:\ds\value) of three times was as threshold value of abdominal construction(TVAC). The [minimum](app:\\ds\\minimum) of injected water volume, which induced rats back arching (abdomen is apart from the horizontal plane about 1 cm) was recorded in the same way. The [mean](app:\\ds\\mean) [value](app:\ds\value) of three times was as threshold value of back arching(TVBA).

Result:

As shown in Table 2, compared with control group, the TVAC and TVBA were significantly decreased in PI-IBS model group (*P*<0.05). TVAC and TVBA of HXZQ treatment groups and TMT group were significantly higher than that of PI-IBS model group(*P*<0.05), and compared with normal group, there was no statistically significant difference.

Table 2 The TVAC and TVBA of rats in each groups（n=8）

| Group | TVAC(ml) | TVBA(ml) |
| --- | --- | --- |
| control | 0.96±0.10 | 1.39±0.14 |
| PI-IBS model | 0.78±0.13** | 1.03±0.14*** |
| TMT | 0.93±0.11## | 1.34±0.12### |
| low-dose of HXZQ | 0.90±0.10# | 1.29±0.15### |
| middle-dose of HXZQ | 0.91±0.12# | 1.33±0.11### |
| high-dose of HXZQ | 0.94±0.08## | 1.36±0.12### |

Values given as mean±standard deviation. ***P*<0.01, ****P*<0.001 vs. control group；#*P*<0.05，##*P*<0.01，###*P*<0.001 vs. PI-IBS model group.
